# Navigating the Alcohol Treatment Pathway: A Qualitative Study from the Service Users' Perspective

**DOI:** 10.1093/alcalc/agv027

**Published:** 2015-03-30

**Authors:** Helen Gilburt, Colin Drummond, Julia Sinclair

**Affiliations:** 1 Addictions Department, Institute of Psychiatry, Psychology and Neuroscience, King's College London, London SE5 8AB, UK; 2 University Department of Psychiatry, Clinical and Experimental Sciences, Faculty of Medicine, University of Southampton, Southampton, UK

## Abstract

**Aims:**

Provision of effective treatment for dependent drinkers has been identified as a priority in England yet evidence suggests that access is problematic and there are low levels of retention. This qualitative study explores how the alcohol treatment system is experienced by service users, identifying barriers and facilitators that influence treatment outcomes.

**Methods:**

A total of 20 semi-structured face-to-face interviews were conducted with patients from community alcohol treatment services in three London boroughs in 2012. Interviews were undertaken one year after initially entering treatment. A thematic analysis was conducted, with the results further abstracted to relate them to specific aspects of the treatment journey.

**Results:**

Patients journeys were characterized by a perceived lack of control leading to help-seeking, with treatment outcomes influenced by an individuals' self-efficacy and the capabilities and skills of staff in actively engaging and supporting patients on the journey. A focus of services on the detoxification process and fragmented care pathways impacted negatively on engagement.

**Conclusions:**

Current alcohol care pathways require significant levels of motivation and self-efficacy to navigate that few patients possess. Pathways need to better reflect the capacity and capabilities of patients to be successful in supporting recovery.

## INTRODUCTION

The cost of alcohol misuse to society in England is estimated at £21 billion a year, with £3.5 billion spent on healthcare and a further £18 billion incurred as a result of alcohol-related crime and lost productivity due to alcohol (Home Office, 2012). The provision of effective treatment for dependent drinkers is a government priority to reduce these costs (Department of Health, 2012).

Internationally most treatment approaches comprise three common stages: detoxification to minimize withdrawal, rehabilitation and maintenance (International Centre for Alcohol Policies, 2005). In the UK treatment is focussed on managing dependence primarily through community agencies providing detoxification and structured psychological interventions; and residential agencies providing inpatient detoxification and rehabilitation (Rose *et al.*, 2011). However, a national needs assessment in England estimated that annually only 6% of those with alcohol dependence access specialist treatment (Department of Health, 2005). Amongst those referred, there are high rates of non-attendance (Mitchell and Selmes, 2007) and less than 70% complete a treatment programme (Passetti *et al.*, 2008). This situation is not unique to England (Capoccia *et al.*, 2007) and improving the effectiveness of alcohol treatment services has been identified as a priority by a number of other countries (National Institute on Alcohol Abuse and Alcoholism, 1997; Pearson, 2011; Shield *et al.*, 2013).

A key element of effective service provision is engagement and retention in treatment. Studies of individual factors which predict initial engagement highlight the role of demographic variables (Weisner, 1993), and person-related characteristics including; prior experience of treatment, symptoms of problem drinking and the social and health consequences of drinking (Hajema *et al.*, 1999). These contribute to the experience of emotional distress, recognition of need and perception of, whether treatment will be effective, or not (Saunders *et al.*, 2006). Similarly, severity of dependence, employment status, marital status, depression and coping style, alongside having clear treatment goals and perceived benefit from treatment have been shown to be positively related to retention (Fiorentine *et al.*, 1999; Mertens and Weisner 2000; Kohn *et al.*, 2002). However with organizational variations serving as a greater predictor of outcome variability, treatment models also play a central role in effectiveness (Simpson, 2004).

Both the structure and context of services have been demonstrated to influence engagement. A lack of wider screening and support in primary care, limited awareness of available services, appointment based systems and limited opening times are each cited as barriers to treatment (Naughton *et al.*, 2013). Subsequently, speed of access, ability to provide individual attention, size of therapy groups and the physical environment of the service, in addition to its location and ratio of medical to non-medical staff have been found to contribute to treatment completion (Stark, 1992; Hoffman *et al.*, 2011). There is little evidence to support the impact of treatment modality but the relationship between the therapeutic relationships and treatment outcome is well evidenced (Najavits and Weiss, 1994; Marsh *et al.*, 2010). In the treatment of alcohol dependency, Ritter *et al.* (2002) found that the degree of perceived therapist expertize and empathy were significantly associated with positive treatment outcomes, and the extent to which self-efficacy and coping skills were acquired was correlated with a perception of therapist as empathic, congruent and displaying high regard for them.

The effective delivery of treatment represents an iterative relationship between client and treatment programme (Simpson, 2004). However, a qualitative study of alcohol treatment services in the UK found disparities between how quality is defined by patients and providers (Resnick and Griffiths, 2012). Furthermore, with services commonly provided by a number of different agencies with varying treatment approaches, the organization of provision including choice of services, allocation of funding and gate-keeping criteria can further influence pathways into services, quality of treatment and completion (Shepard *et al.*, 2002; Webb *et al.*, 2008; Resnick and Griffiths, 2012). Building an understanding of how these factors interact is important to improving the effectiveness of provision.

Using the perspectives of service users to understand the impact of the service delivery models has played an important role in a number of areas of healthcare (Smith and Ross, 2007). This study seeks to build on that tradition to explore the experience of individuals seeking help for alcohol dependence and their journey through the alcohol treatment system as a means of better understanding the role that personal, treatment related and organizational factors interact in the pathway to recovery.

## METHODS

### Sample and recruitment

Participants were recruited as part of a pilot randomized controlled trial of assertive community treatment for alcohol dependence compared with treatment as usual. Inclusion criteria were: age over 18; contact with the participating NHS community addiction services in the past 5 years; and a diagnosis of alcohol dependence. Only those randomized to the treatment as usual arm of the trial were eligible to take part in this qualitative study. A full description of treatment as usual is provided in the trial protocol (Gilburt *et al.*, 2012).

A sampling framework was used to recruit a maximum variation sample (see Table 1) ensuring a range of perspectives rather than a means for comparison. Individuals were categorized according to information collected in the trial, including engagement with alcohol treatment services, alcohol consumption in the previous 90 days and severity of alcohol dependence.

**Table 1.** Sampling framework used to identify participants

1	Individuals who had engaged in the treatment offered and had significantly reduced their alcohol intake or were abstinent
2	Individuals who had not engaged in treatment or disengaged at the early stages of treatment and had significantly reduced their alcohol intake or were abstinent
3	Individuals who had engaged in treatment and were drinking at the same level
4	Individuals who had not engaged in treatment or disengaged at the early stages of treatment and were drinking at the same level

### Ethics

Ethical approval was obtained from the National Research Ethics Committee (08/H0801/113). Following completion of the main study follow up questionnaires 12 months after entering the trial, potential participants were invited to discuss their experiences of treatment over the past year. Those who agreed provided written informed consent. As a token of appreciation participants were given a £10 food voucher.

### Data collection

All qualitative interviews were conducted by HG. Participants were given the choice of time and location. The majority opted to take part at the same time as completing the trial with most interviews taking place in their place of residence. A series of open-ended questions guided participants in describing their journey and experiences from the point of seeking help one year previously to the present. Prompts encouraged descriptions of the different types of services used, what had been helpful and unhelpful, if there was anything that could have been done differently, and external factors which were perceived to have influenced their drinking during this period.

### Analysis

Interviews were transcribed verbatim facilitating familiarization and immersion in the data. The analysis process was data driven, focussed on the semantic level and based on the framework for thematic analysis outlined by Braun and Clarke (2006). Two members of the research team (HG and JS), analysed a number of transcripts independently, discussed findings and then HG conducted the initial coding of the transcripts. This was an iterative process which involved re-reading the transcripts to ensure the groundedness of the emerging codes.

After preliminary analysis, the emerging coding framework was discussed between all authors in a process of triangulation to obtain different perspectives on the data. Where questions were raised, the transcripts were re-examined and the coding framework adjusted to reflect new insights and understandings of the data. The qualitative analysis software NVivo was used to organize and document the analysis process.

## RESULTS

Twenty participants were recruited, including eleven men and nine women. They varied in age (22–55 years; *x* = 40; s.d. = 8) and severity of dependence at the point of help-seeking (Severity of Alcohol Dependence Questionnaire score 10–54; *x* = 35, s.d. = 14) and all but one was White British. Four participants met the criteria for each of categories 1 and 2, and six participants for each of categories 3 and 4 (see Table 1).

Seven themes emerged from the data which pertain to participant's experiences of alcohol treatment services. They comprize:

- Recognizing tipping points
- Treating alcoholism and working with drinking
- Characteristics of active engagement
- The role of self-efficacy
- Making sense of alcohol dependence and being an alcoholic
- Journeying around the treatment system
- The role of 12 step groups.

In this paper these themes are abstracted further to relate them to specific aspects of the journey through alcohol treatment services.

### Seeking help and entering the system

Participants described many problems in their lives, which they related to the need to tackle their drinking or seek help. This included loss, both actual and feared (e.g. ‘I lost my home’; ‘I will lose my job’; ‘I was always an active person … I just felt myself slipping’ [loss of identity]); perceived role as a caregiver (‘I need to be a parent’); experienced impact on family (e.g. child removed; grandparents openly crying); as well as the deleterious impact of the alcoholic ‘lifestyle’.

In addition, the embodied experience of alcohol dependence was raised by almost all participants. The descriptions of physical symptoms such as sickness, diarrhoea, tiredness, sweating, breathlessness and pain were vivid, extensive and it is clear that they had a powerful impact on individuals.

The role of the family was highly influential in help-seeking by confronting participants about their drinking or giving ultimatums, but more often in providing a mirror by which the behaviour and impact of the participants alcohol dependence was reflected back to them. In some cases family members were the driving force for treatment entry, however this alone was not necessarily conducive to recovery.

… before it was always for someone else. I'd do it for the kids, do it for my partner, do it for my family. … So I put me first and I've found that was my recovery, that's how I had to get to that place. #026

The decision to seek help was characterized by reaching a point of being ‘out of control’. This referred to a perceived lack of agency, often resulting from the culmination or increased frequency of several factors (e.g. regular seizures, drinking every day); and in some cases the impact of a new (e.g. nosebleed; ‘wetting myself’) or significant event (e.g. contact with police, hospital or social services).

Although some described the process of seeking help as a clear linear pathway, for many others it was much more complex. Participants describe accessing treatment from a number of services and sites, across primary, secondary and tertiary health care, from different sectors (e.g. social care, housing and health), both formally and informally, often at the same time. Sometimes the complexity reflected the nature of the fragmented services provided, but elsewhere it highlighted a participant's chaotic pattern of engagement resulting from opportunistic or volitional encounters with a multitude of services.

… one of the days when I was coming home from (NHS community alcohol service), I thought I'm going to pass (third sector alcohol service) so I went in there … and they made an appointment for me. So I was sort of skipping backwards and forwards to them. #127

Identification of alcohol problems by non-specialist staff, and staff from specialist alcohol services in non-specialist settings (e.g. GP surgeries, acute hospitals), could be beneficial in facilitating access to specialist care. However where there was a lack of integration with specialist providers and a number of participants received interventions (e.g detox) in the absence of an adequately formulated post-treatment plan or contrary to an existing one. Although initially perceived as beneficial by participants in offering timely access, in hindsight they described being insufficiently prepared for treatment and lacking on-going support.

### Characteristics of active engagement and the role of self-efficacy

The nature of therapeutic relationships and the quality and quantity of structural supports available during treatment affected how well people felt their recovery was facilitated. A core element of this was the ability to maximize and support self-efficacy.

Many of the elements of care that participants noted to be of value in their interaction with practitioners were characteristic of the universal features of therapeutic relationships. Participants highlighted examples of acts of advocacy, kindness, compassion and staff ‘going beyond’ their basic role demonstrating to participants that staff ‘wanted the best for me’, and ‘they must think I can do it’, championing the individual, building trust, confidence and motivation. The importance of practitioners being assertive was raised by several individuals. Phoning and chasing people up, and an assertive manner of interaction that challenged individuals was highlighted as particularly important. This was less about dictating treatment, but rather addressing issues of motivation and being honest and overt about the process and requirements of treatment.

I think [alcohol service], you know they were there for me all the time. They didn't put up with no bull. You know, it's like you can try and pull the wool over someone's eyes but they were there, just like frank. … Like I need someone to tell me what to do. #006

The converse; feeling that you were ‘being judged', staff not listening to or believing you, being dismissed as ‘unmotivated' for missing an appointment, people being rude, all fed into a participants' internalized negative identity as an alcoholic, low self-esteem and sense of poor self-efficacy.

Participants recognized that services wanted people to be ‘seen to be helping yourself’ and this contributed to the process of recovery, supported by services ‘pointing you in the right direction’, offering timely support, advice, encouragement and robustly keeping patients to task. Where participants describe taking a passive role, or when treatment options were more prescribed, the rationale and conditions for treatment were often insufficiently understood or conflicted with their own explanatory models and had a negative impact on engagement. While this led many to drink, it is notable that for a couple of people it triggered the individual to take control.

… so what you are saying is, I'm going to go into a group and all I am going to do is talk, but what I want is drugs. I don't want to talk about it, I can talk till the cows come home. So I went home and I thought but this is madness, talking to people doesn't work for me. And I thought, I've got to do this on my own anyway so even if I go into a group session, no matter where I go or who I talk to, I've still got to go home and be the person that says, look I still can't do this, I can't drink. So nobody is going to do this for me, only me. #219

The concept and process of reducing alcohol consumption was described positively by a large number of participants. A significant part of the value ascribed to it was the feeling of agency and regaining control it provided, together with a great sense of achievement.

I never thought I'd be able to go a few days without drinking, you know it was hard enough to do a day without drinking. So you know I go round to [girlfriends] flat, … and I drink a lot of herbal tea. #109

Participants emphasized the need for frequent contact on which relationships could be built and in maintaining motivation and continuity. Some community programmes were seen to offer too few and infrequent sessions. This limited the support available during and after treatment and provided insufficient structure and detachment from the pre-treatment lifestyle. Residential programmes were generally described as providing good levels of continuity, although both staff and patient turnover could disrupt the group dynamics that had been established, resulting in a loss of trust and feelings of safety.

The challenge of attendance was potentiated practically by the drinking behaviour of participants and by feelings of shame associated with continued drinking and relapse: missed appointments were cited as common. This commonly resulted in a verbal or written letter of discharge from the service. The effect of drinking on disengagement was most prominent in rehabilitation services as a result of abstinence being a requirement for attendance.

I didn't go back to (community programme) … because I got caught drinking and it's embarrassing #006

And because I didn't make my appointments they used to write like, ‘you can go. If you want to reassess you can [return], after like four months.’ #123

### Concepts of alcohol dependence and treatment

Treatment was described as focussed on dependent drinking and achieving abstinence. Participants' concepts of dependence varied considerably; including volume and/or strength of alcohol consumed (vodka and super-strength drinks perceived as indicative of more severe problems); and a need, as opposed to a want, to keep drinking. These concepts influenced access to services and were reflected in treatment provision. A number of participants stated treatment was for when ‘you're really, really ill’ or when all else fails, while others reported being told by treatment staff that they ‘weren't drinking enough’ or their pattern of drinking did not reflect a requirement for treatment. This led to some participants to delay treatment, and others who sought treatment early being turned away.

… he said, we can't help you. You don't drink every day, you're a binge drinker; … And after that I just thought well, ‘stuff the lot of you’ basically. Because, alright I'm a binge drinker so what have I got to do, go out and drink every day and make myself worse in order to get some kind of help. #026

Cutting down was seen by many as an integral step to regaining control of drinking and/or attaining abstinence. Reducing consumption was often supported in principle by services, but when staff conceived it an outcome rather than a process it led to individuals being told it was not an option. In addition, conflict arose between participants' perception of cutting down as a logical pathway to tackling excessive drinking and advice from staff in some cases to ‘keep drinking’ in order to prevent fits, withdrawal and ensure access to treatment.

Treatment was often described by participants as ‘getting a detox’ in a somewhat matter of a fact way. This was reinforced by the largely functional role that alcohol keyworkers were seen to take; doing assessments, providing set advice, performing tests and setting up interventions. However there was a degree of confusion around what this actually meant, from perceptions that the medication would essentially ‘cure’ them, to detoxification as a process. While many individuals recognized the limitations of a medically assisted detoxification, it led to disappointment for others, and two participants describe being ‘denied treatment’ on the basis of being refused a medical detoxification, even though at the time they were not alcohol dependent.

Having a clear understanding of what to expect post-detoxification, learning about alcoholism and the impact of alcohol, and receiving support to develop practical skills to deal with real world situations in which they would encounter drink were identified by participants as important both in preparation for treatment and rehabilitation after. Despite this, treatment services were often seen to focus predominately on physical dependence, while some experienced rehabilitation groups as lacking focus or endlessly talking about drinking, providing little evidence of how to succeed at being abstinent within the contexts of their lives and the societal norms in which they functioned.

[Treatment service] was all right but as I say you can't be wrapped in cotton wool there. You go there and you come out of there. Then you're in [town] and you think, well now what. #015

Many participants saw their alcohol dependence as a consequence of broader challenges they faced. Talking therapies were highlighted by almost half as important in supporting recovery, although access was described as poor. One to one counselling was seen as different in purpose from key-working and characterized by talking, in particular, about how you feel and dealing with complex, often psychological issues which were described as being ‘at the heart of the matter’. Many of these were stressors which participants identified prior to help-seeking, however the treatment process itself led others to reflect on their identity, and the negative impacts of their drinking, a process described by one man as ‘raw and intense’. Peers, especially at similar stages of recovery and motivation, could be important in facilitating personal learning through sharing different stories and views, and as a source of support and understanding, but group settings could also inhibit disclosure. For many overall, it was the facilitation of self-reflection to develop a deeper personal understanding and individual narrative which proved key.

More than half of participants had experienced a 12-step programme. Twelve-step programmes were predominately spoken about as outside usual treatment services or within the context of rehabilitation. Many rehabilitation programmes used the model and attendance at AA groups was integrated or encouraged. Programmes based on other theoretical models however, often did not support attendance of AA. Those who spoke positively about 12-step programmes described many of the elements of value described previously including structure, frequency of sessions and peer support. A number of people described being ‘pushed to AA’ and overall there was an impression that AA was second class to ‘treatment’ or not part of the legitimate treatment services available.

last year in treatment they were quite against the 12 step programme and you weren't allowed to go to meetings, … I've obviously realised, having the 12 step in my life now, I needed that structure. I needed a set of principles and a set of rules to live by. #120

I told her that I had started drinking again. … She said it's up to you to decide what you want to do, and basically, that was it. All she kept trying to push at me was AA really. #214

### The role of agency in navigating treatment and recovery

Participants described the significant personal investment and determination that was required to access services and continue in treatment; including attendance of multiple scheduled appointments across different services. In some cases, these were described as being in limited timeslots and at inconvenient times.

I have never missed an appointment, never, but it's just, ok we'll pass you on to this place, or we'll pass you onto here. And I've gone there, gone there. Same old questions, same things. Nothing seems to have [been] put [in place], that's why I've been driving [keyworker] mad to say ‘come on man’. #127

A lack of role definition and role boundaries in services, overlap in treatment provision and poor service integration resulted in a number of unforeseen consequences. Participants were often unclear about who they were engaged with and their role. It was not uncommon for participants to describe several people in different agencies as taking a lead role in their alcohol treatment at the same time. As a result some described receiving conflicting information, or having to complete duplicate assessments at different services. A number of individuals bounced between different services accessing different services at different times, while others exploited the situation as a means of optimizing their access to treatment options, accessing several of these services at the same time; in practice both often led to fragmented engagement and delays in treatment.

Participants' accounts of self-efficacy were underpinned by a perceived balance of responsibility between themselves and the services from which they were seeking help. This balance was described as being influential on outcomes. A number of participants felt that more often the balance of responsibility lay with them, a view that was in part informed by previous experiences of services. For two people the belief that treatment services were not going to help drove them to achieve abstinence independently. However for the majority it resulted in early disengagement or situations in which considerable assertiveness on their part resulted in access to treatment in the short term, but disengagement later. In all these cases, individuals expressed high levels of resentment.

So, I knew I had to do something but, and I knew that I couldn't get the help that I wanted anywhere so, it was trying to work out a plan for myself. #026

And they said to me, they'd get in touch with me here. But they never got in touch … So I thought, well they've not got in touch with me, what's the point of me trying to get in touch with them. If I'm trying to get myself help and they're ignoring me … #132

A sense of self-efficacy could be experienced as empowering but was balanced against considerable pressure associated with the inherent responsibility. It is perhaps not surprizing therefore that with the focus on self, both as the cause of alcohol dependence as well as the primary moderator of change, over half of all participants reported feelings of failure and self-blame ‘And I was doing good, I just screwed up.’ Both the interviewer and an independent transcriber noted a pervasive sense of hopelessness in the interviews, all having had previous contact with services for alcohol dependence and the perceived overwhelming onus of responsibility on individuals.

I could have been more assertive, like doing things, or like if I got a bloody runny nose, don't call up and say I'm ill and I can't make an appointment. If I waited for someone to help me I would be waiting a long bloody time, I've got to do it myself. #006

I've not really had, it's my own fault. I've not, I've known there's treatment out there, but people get to the stage where you think they think, and you get the vibe, if you can't be arsed, then I am not arsed. #131

## DISCUSSION

The experiences and drinking outcomes of the patients interviewed in the study were varied but overall they highlight a picture prior to treatment of lives that were completely dominated by drinking, deleteriously impacting on family, work, their health and identity; such that the final trigger for help-seeking is one of feeling completely out of control. The perceived role of services is where appropriate support is provided to address the physical and psychological impact of alcohol, and build the underpinnings of a life without it.

Length of service contact is the single best predictor of post-treatment recovery status (Hubbard *et al.*, 1989, 2002; Simpson *et al.*, 1997; Simpson and Brown, 1999) yet treatment retention rates are low. Participants in our study described a number of barriers to engagement including having to attend numerous appointments, often infrequent contact with services and engagement with multiple workers and agencies in order to obtain treatment. Given the characteristics of alcohol dependence including disorganization (Bauer, 1982), decreased ability to make judgements, cognitive impairment (Oscar-Berman *et al.*, 1997) and fluctuating motivation these barriers present a significant challenge for the continuing attendance of individuals. The additional requirement of abstinence, either in attending appointments, or for engagement with rehabilitation services further precludes engagement. With failure to attend and relapse commonly described as indicators of poor motivation, treatment delivery systems may fail to take sufficient account of the nature and impact of alcohol dependence, as a result making unrealistic demands on individuals in order to receive treatment.

The importance of self-efficacy permeated participant's experiences. Self-efficacy is a key factor in the process of change (Connors *et al.*, 2001; Simoneau and Bergeron, 2003) and a predictor of sustained recovery (Best *et al.*, 2010). Health care professionals play an important role in supporting and building self-efficacy. Participants' accounts highlight a number of mechanisms by which this can be achieved including building positive therapeutic relationships with staff, use of assertive engagement techniques, honest and overt discussions about treatment and providing advice and encouragement. As a requirement of structured treatment (National Treatment Agency for Substance Misuse, 2006) care planning provides a vital tool for engaging individuals to identify key components in their recovery and building their sense of ownership and efficacy. However, the majority of participants in this study were unclear about the overall plan for treatment and recovery, with recognition of a plan often only extending to the immediate service with which they were engaged or to broad principles of treatment such as detoxification and rehabilitation. Using care planning as a process rather than a tool and focussing on sequential stages of recovery may limit it validity with individuals (National Treatment Agency for Substance Misuse, 2006) and impact negatively on self-efficacy.

One of the limitations of being able to plan care across the alcohol treatment pathway is the number of different services and boundaries between these services. Our findings show the negative impact that this can have. Participants highlighted a range of different routes into treatment with the subsequent treatment journey often characterized as fragmented, with input from a number of different staff in different settings and an overall lack of clarity around the role and remit of each. Targeting interventions through multiple access points can improve choice, accessibility and equity (Richards, 2000) but can fragment care without appropriate co-ordination. Overlap and gaps between services resulted in patterns of engagement that are inefficient and experienced as demotivating and disempowering. These experiences are further influenced by the poor integration of different models of treatment throughout the pathway, including medical, psychological and spiritual which served to inadequately acknowledge some fundamental components of addiction from the outset and devalued others. This fragmentation is not unique to the United Kingdom and has been identified as major barrier to effective treatment in the United States and Australia (National Institute on Alcohol Abuse and Alcoholism, 1997; Pearson, 2011). It may be reduced through augmentation of existing practice with ‘assertive linkage’ to services where staff make active attempts to ensure engagement (Best *et al.*, 2010), or case management (McLellan *et al.*, 2005). Additionally the allocation of clear responsibility around access and provision with responsibility for funding across the care pathway (Alcohol Concern, 2013) provides a mechanism for integrating both services and models of treatment.

The treatment system described is one focussed on the detoxification process from alcohol dependence. Our analysis identified that as patient's concepts of dependence are often contrary to that of services, many find themselves unable to enter treatment when they recognize a need for it, or having to wait until they meet service defined criteria. Furthermore, although the process of reducing drinking was described positively in terms of engagement and gaining self-efficacy, the focus on achieving and maintaining abstinence often did not support this or offer a pathway back into services after a lapse back to drinking. Harm reduction is a core element of UK drug treatment programmes but its application in specialist alcohol services is disputed (Luty, 2006). Goals of reduced consumption can be beneficial in arresting escalating patterns of drinking (Ambrogne, 2002) and attract and retain individuals who are ambivalent or who would otherwise not seek or participate in therapy (Heather, 2006; Sinclair *et al.*, 2014). Such strategies may be beneficial in facilitating early access to services while increasing the self-efficacy of those in the initial stages of treatment.

The experiences described by participants in this study encompass a variety of engagement and drinking outcomes but cannot be representative of all service provision. However, that many of their experiences reflect issues highlighted in the wider literature suggests that they are not unique, and furthermore that they are illustrative of long-term continuing problems. The conceptual framework for alcohol treatment systems proposed by Babor *et al.* (2008) highlights the importance of treatment provision, treatment policies, system qualities and social capital on the effectiveness at a population level. While there is evidence of good practice in the provision of care and services, in practice the underlying framework of delivery in England, including treatment policies, programmes and the care pathway at a service level, in addition to the limited provision for long-term recovery means that only those who can demonstrate considerable and sustained levels of motivation and self-efficacy are likely to succeed.

## FUNDING

This work was supported by the Medical Research Council [grant number G0701818]. Colin Drummond is partly funded by the NIHR Biomedical Research Centre for Mental Health at South London and Maudsley NHS Foundation Trust and King's College London and partly funded by the NIHR Collaborations for Leadership in Applied Health Research and Care South London at King's College Hospital NHS Foundation Trust. Funding to pay the Open Access publication charges for this article was provided by Medical Research Council as part of the block grant research councils allocate to each institution in the UK for open access publication.

## CONFLICT OF INTEREST STATEMENT

None declared.
